# Late diagnosis of multidrug-resistant tuberculosis in a child at Dr George Mukhari Academic Hospital, Ga-Rankuwa, South Africa: A case report

**DOI:** 10.4102/ajlm.v8i1.783

**Published:** 2019-07-29

**Authors:** Bakani A. Siwele, Ndivhuho A. Makhado, Matodzi T. Mariba

**Affiliations:** 1 Department of Orthopaedics, Sefako Makgatho Health Science University, Pretoria, South Africa; 2 National Health Laboratory Service – Dr George Mukhari Tertiary Laboratory, Department of Medical Microbiology, Pretoria, South Africa; 3 Department of Microbiological Pathology, Sefako Makgatho Health Sciences University, Pretoria, South Africa; 4 Global Health Institute, University of Antwerp, Wilrijk, Belgium; 5 Department of Biomedical Sciences, Mycobacteriology Unit, Institute of Tropical Medicine, Antwerp, Belgium

**Keywords:** spinal tuberculosis, extrapulmonary tuberculosis, multidrug-resistant tuberculosis, laboratory diagnosis, radiological improvement

## Abstract

**Introduction:**

South Africa has one of the top ten tuberculosis burdens in the world, only lagging behind countries with significantly larger populations. Increased awareness of extrapulmonary tuberculosis, specifically spinal tuberculosis, is necessary, because of the HIV epidemic.

**Case presentation:**

This report describes the case of a 9-year-old male patient who was suspected of having multidrug-resistant (MDR) tuberculosis, based on failure to recover clinically and radiologically after 6 months on first-line anti-tuberculosis treatment. Pus samples were sent to an accredited academic laboratory for histopathology, microscopy, culture, line-probe assay (MTBDR*plus* assay) and phenotypic MGIT 960 drug susceptibility tests. Second-line MDR tuberculosis treatment was introduced. Clinical, radiological, physical processes and more laboratory tests were conducted to document whether or not there was improvement in the patient.

**Management and outcome:**

After laboratory diagnosis of MDR tuberculosis, the patient was started on MDR tuberculosis treatment. The patient started improving remarkably after the introduction of anti-tuberculosis treatment and rehabilitation, although he also required surgery to stabilise the spine. Neurological improvement was observed in the patient and he fully recovered.

**Discussion:**

Although the diagnosis of spinal MDR tuberculosis may not be achieved easily by culture, the well-known gold standard method of tuberculosis diagnosis, it is of great importance to rapidly initiate an effective anti-tuberculosis treatment of drug-resistant strains to reduce the deformity of the spine.

## Introduction

Globally, tuberculosis is the most common infectious disease and is responsible for a high rate of morbidity and mortality when not properly managed.^1^ Co-infection with HIV and tuberculosis, and the increasing emergence of drug-resistant (DR) strains of tuberculosis, especially multidrug-resistant (MDR) tuberculosis and extensively drug-resistant (XDR) tuberculosis, present a major threat to effective tuberculosis control.^2,3^ Not only is South Africa one of the top 10 countries in the world for prevalence of tuberculosis, but it also has the highest number of patients with DR tuberculosis in the world. However, it does lag behind countries with significantly larger populations, such as India and China.^4^

Tuberculosis is a highly infectious disease of the lungs. Pulmonary tuberculosis, caused by the *Mycobacterium tuberculosis* complex, can also affect other body sites as extrapulmonary tuberculosis; spinal tuberculosis is a frequently encountered form of extrapulmonary tuberculosis.^5,6^ Despite the availability of anti-tuberculosis therapy, the vertebrae and spinal cord often have a delayed diagnosis leading to devastating, irreversible complications, for example paraplegia.^7,8,9^ Furthermore, it has been proven that half of extrapulmonary tuberculosis cases have been previously reported, and delayed diagnosis may further enhance the risk of transmission of tuberculosis via contacts with more people, as well as compromising healthcare workers.^10^

In this case study, factors associated with clinical aspects, radiology and late diagnosis of MDR tuberculosis of the spine and its clinical outcome are reported.

## Ethical considerations

Oral consent was obtained from the patient’s parents along with assent from the patient and ethical clearance from the Sefako Makgatho Health Sciences University’s Research and Ethics Committee (SMUREC/M/147/2017:J) for the publication of this case study, including any images in any abstract or publication. All personal identifiers were anonymised for confidentiality before data processing was performed. There were no patient identity links to the radiological images used.

## Case presentation

A 9-year-old male patient with a history of backache was referred from a district hospital to the Dr George Mukhari Academic Hospital, Gauteng province, South Africa, due to severe upper back pain and gibbus (kyphosis) deformity that emerged over 8 months prior to admission. He had no history of tuberculosis contact or constitutional symptoms (cough or fever, loss of weight or loss of appetite).

Upon physical examination at admission, the patient was ambulatory but limping, had tenderness at the gibbus area, with pain in the waist. He was cooperative and appeared to have a normal skin condition. There was no Bacillus Calmette-Guerin scar observed. The neurological examination was normal and intact.

**FIGURE 1:**
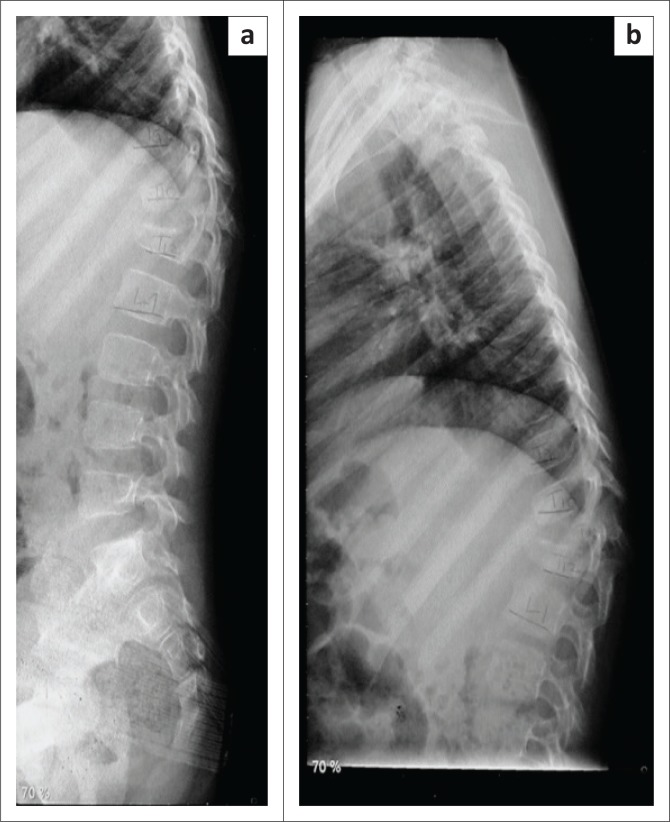
X-rays showed (a) L1 upper end plate and (b) T12 lower end plate destruction with peri-vertebral shadow.

X-rays revealed kyphosis at the bottom of the thoracic spine (T12 vertebra) and the first vertebra of the lumbar spine (L1 vertebra). His T12 had vertebral destruction with preserved posterior elements. The L1 upper end plate and T12 lower end plate destruction with a peri-vertebral shadow were indicative of an abscess (Figure 1).

**FIGURE 2:**
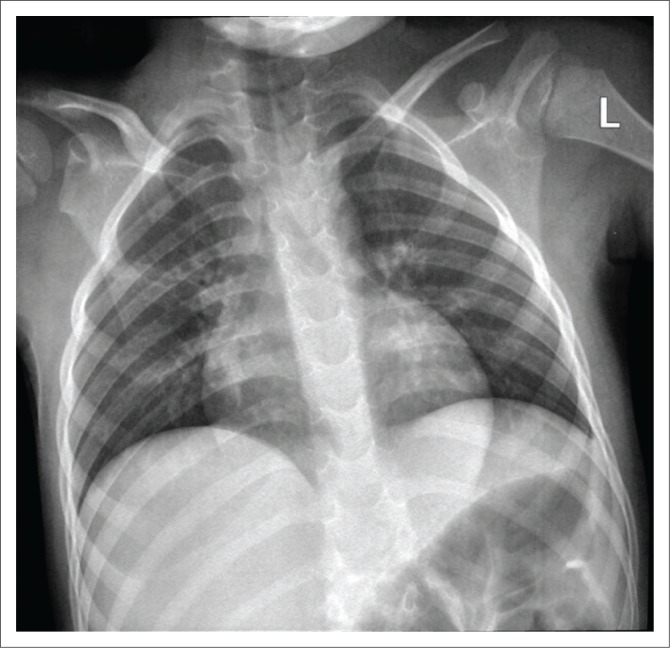
Plain X-ray demonstrating unremarkable features of the chest.

Plain radiography of the chest was unremarkable (Figure 2). Furthermore, the haemoglobin was low (11.5 g/dL), with a normal-range white blood cell count and an elevated C-reactive protein level of 18 mg/L. The erythrocyte sedimentation rate was a bit high at 20 mm/hour; alkaline phosphatase was 211 U/L. The alanine aminotransferase (19 U/L) and aspartate aminotransferase (31 U/L) were within the normal ranges. A serology test for HIV enzyme linked immunosorbent assay was negative. A superficial pus swab microscope analysis was acid-fast bacilli-negative when stained with auramine-O. An MGIT 960 culture was also negative after 42 days.

**FIGURE 3:**
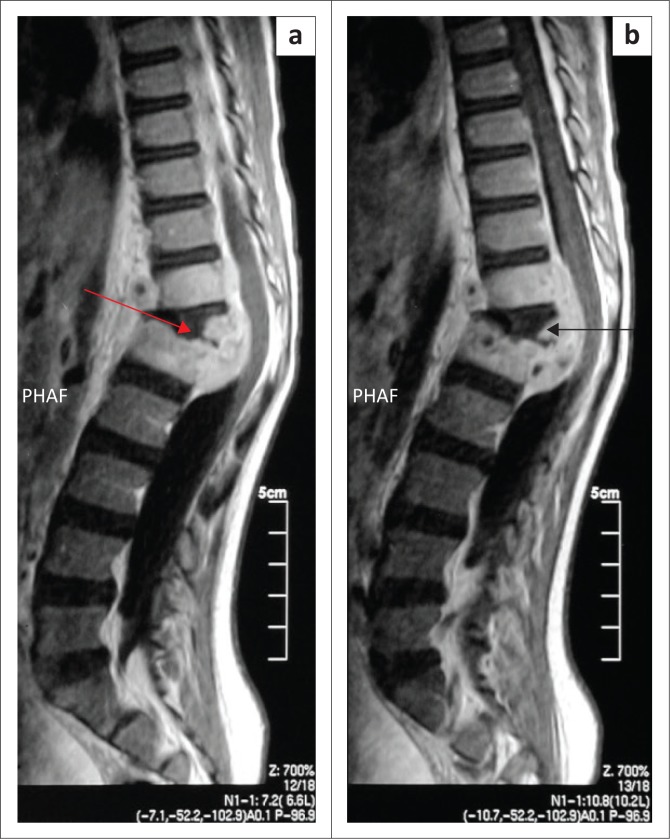
The magnetic resonance imaging of the spine revealed (a) kyphotic deformity and (b) pre-vertebral fluid collection at T12/L1 extending to the epidural space.

Magnetic resonance imaging of the entire spine revealed features suggestive of tuberculosis infection (Figure 3). There was kyphotic deformity and pre-vertebral fluid collection at T12/L1 extending to the epidural space. The fluid collection was low on T1, high on T2 and became enhanced post-contrast. There was also an abnormal signal of L1 and T12, as well as associated involvement of the superior end plate of L1. The T12/L1 intervertebral disc was not clearly delineated with the distortion of normal anatomy. Spinal canal stenosis was observed at the level of kyphotic deformity with cord compression, although there was spinal cord edema. Posterior elements were preserved and facet joints were normal (Figure 3).

**FIGURE 4:**
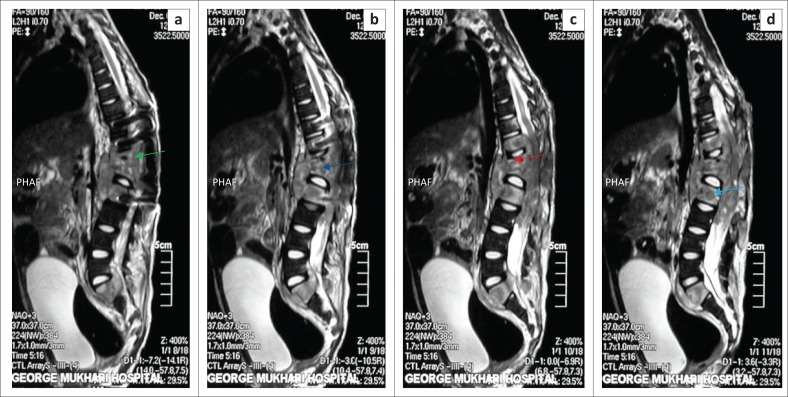
The magnetic resonance imaging of entire spine revealed (a = green arrow) an increased kyphosis, and (b = dark blue arrow) a paravertebral abscess. Furthermore, it demonstrated a complete vertebral destruction of lower and upper end plates of T12 (c = orange arrow) and L1 (d = light blue arrow).

A second set of the magnetic resonance images of the entire spine, which was done eight months later, revealed progression of the disease (infective process), increased kyphosis, and a paravertebral abscess, as well as complete destruction of lower and upper end plates of T12 and L1 with cord compression (Figure 4).

## Management and outcome

The patient was admitted and subsequently started on empirical first-line anti-tuberculosis treatment as recommended by the World Health Organization, as well as other supportive treatment, including thoracic lumber spinal orthosis. The patient did not improve clinically after 1 month of administering the empirical treatment.

**FIGURE 5:**
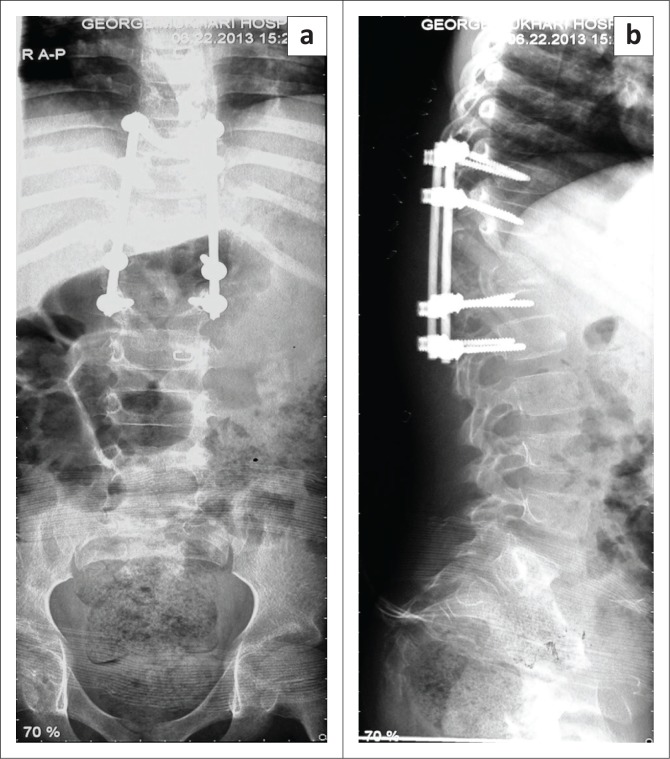
X-rays revealed intact spine post-surgery and decompression (a and b).

Non-operative treatment included a thoracic lumber spinal orthosis brace, analgesia and first-line tuberculosis treatment to prevent further collapse and back care. The patient’s mother consented to operative management, which included spinal decompression and posterior instrumentation, with rods and screws, as well as a biopsy. The patient was neurologically intact post-surgery (Figure 5). The operation was successful.

The pathological diagnosis of the spine (T12/L1) on the vertebral lesion biopsy revealed the presence of chronic necrotising granulomas with multinucleated Langhans-type giant cells. Ziehl-Neelsen staining, used to confirm acid-fast bacilli, was negative. The patient’s first-line anti-tuberculosis treatment, included pyrazinamide, rifampin, isoniazid and ethambutol. The patient’s condition deteriorated despite a total of 6 months of first-line anti-tuberculosis treatment, and the surgical wound started to open leaving space between the sides of the incision (gaping).

**FIGURE 6:**
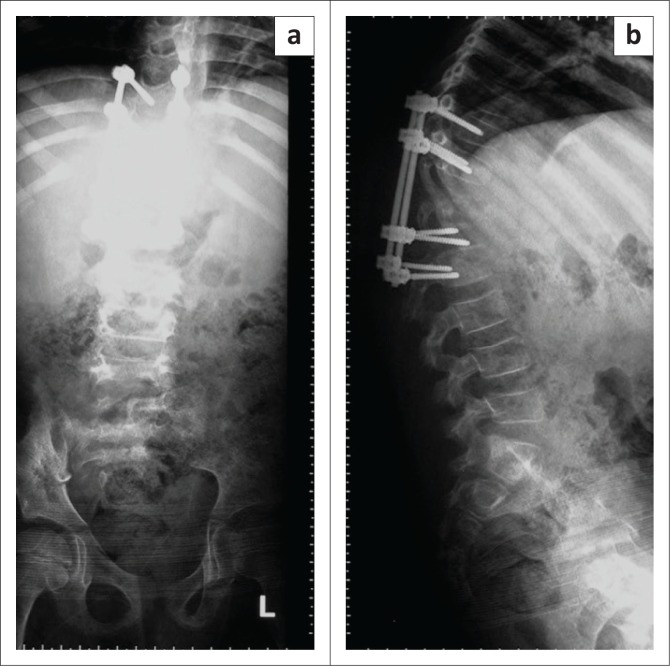
X-rays showing failed implants backing off from the (a) distal part of vertebra (T12/L1) and (b) increased kyphotic deformity of the spine.

X-rays showed failed implants; therefore, the pedicular screws were backed off from the distal part of the vertebra (T12/L1) with increased kyphotic deformity of the spine (Figure 6).

When the patient was on rehabilitation, he developed an abscess on the forehead, which was later drained in the ward, and sent to the National Health Laboratory Services – Dr George Mukhari Academic Hospital tertiary laboratory for further mycobacteriological tests. Fluorescent microscopy of the smear stained with auramine-O was negative for fluorescing acid-fast bacilli. The MGIT 960 (Becton Dickinson Diagnostics, Sparks, Maryland, United States) culture was also negative after 42 days.

The patient was sent to surgery for incision and drainage of the abscess on the gibbus area, where from thick pus was drained and sent for tuberculosis microscopy, culture, line-probe assay (MTBDR*plus* assay, Hain Life Sciences, Nehren, Germany) and phenotypic MGIT 960 drug susceptibility testing. The auramine-O smear results were negative, whereas the MGIT culture was positive after 35 days. The line-probe assay result showed rifampin monoresistance, which was later confirmed by the phenotypic MGIT 960 drug susceptibility testing as rifampin monoresistant, ethambutol resistant and isoniazid sensitive after 9 months of first-line anti-tuberculosis treatment. The second-line MGIT 960 drug susceptibility testing showed that the isolate was susceptible to moxifloxacin and kanamycin. This led to an MDR tuberculosis diagnosis, since tuberculosis caused by rifampin-resistant strains is presumed to be MDR tuberculosis and treated as such (95% of rifampin-resistant strains are MDR^3^). The patient was started on the MDR tuberculosis treatment, including moxifloxacin, ethionamide, amikacin, pyrazinamide, terizidone and pyridoxine; he started improving remarkably on the treatment and rehabilitation. At 15 months, the patient fully recovered and the implants were later removed.

## Discussion

The diagnosis of extrapulmonary tuberculosis is not clinically easy due to the non-specific manifestations of the condition; thus, a high index of suspicion is required to accurately diagnose the condition.^7^ Patients who present with focal or spinal abnormalities, with or without a chest radiograph showing previous or active tuberculosis, should be investigated for spinal tuberculosis. Spinal tuberculosis seems to be due to an arterial or venous route of infection. In the early stages, the infection affects the anterior portion of the vertebra.^5^ Osseous tuberculosis may spread to an anterior type involving the vertebral bodies.^7,11,12^ Commonly, patients with spinal tuberculosis present in the first three decades of life with subtle symptoms, such as backache, malaise, loss of appetite and weight, night sweats and fever, as observed in this study.

In this case, the patient had progressive back pain over several months with localised tenderness of the spine and weight loss. Although signs and symptoms of systemic infection are often missing, severe back pain is the typical presenting symptom of early spinal tuberculosis disease and should encourage further investigation of a definite diagnosis. Symptoms such as fever and weight loss in spinal tuberculosis patients are present in less than 40% of cases and are often not specific.^7^

Common symptoms of spinal tuberculosis include leg weakness (69%), gibbus deformity (46%), pain (21%), and palpable masses (10%).^9^ In this study, the patient had no spinal cord involvement despite the delayed diagnosis. In the advanced stages of spinal tuberculosis disease, a significant proportion of patients present with neurological impairment.^9,13^ Kyphotic deformity is seen in 95% of cases, although the deformity may not develop in the early stages or in less severe cases.^2,14^ Less than 40% of patients present with constitutional symptoms of generalised body malaise, loss of appetite, weight loss, night sweats and fever.^5,7^

The majority of spinal tuberculosis cases can be managed non-surgically. Radical surgery at a younger age, combined with anti-tuberculosis treatment, has been associated with favourable patient outcomes.^15^ The surgical procedures can be done anteriorly or posteriorly with instrumentation. Other studies have reported that about 0.7% of patients can have implant failure following a spinal tuberculosis operation.^12^ In this study, surgery was performed and anti-tuberculosis treatment was given. The patient fully recovered after 15 months.

### Conclusion

In conclusion, drug-resistant spinal tuberculosis cannot be easily diagnosed by gold standard methods. A high index of suspicion followed by clinical examination, radiological examination and laboratory investigations will help to promptly diagnose the condition and allow for effective patient management. With emerging MDR tuberculosis, the osseous abnormality is also at risk of involvement. A diagnosis of MDR tuberculosis of the spine should be borne in mind, if the patient does not respond to the first-line of treatment. Drug-resistant anti-tuberculosis treatment and radical surgery can lead to a reduction of spinal tuberculosis complications such as paraplegia.
